# Nanotechnology-Based Drug Delivery Systems for Photodynamic Therapy of Cancer: A Review

**DOI:** 10.3390/molecules21030342

**Published:** 2016-03-11

**Authors:** Giovana Maria Fioramonti Calixto, Jéssica Bernegossi, Laura Marise de Freitas, Carla Raquel Fontana, Marlus Chorilli

**Affiliations:** 1 Faculdade de Ciências Farmacêuticas, UNESP—Univ. Estadual Paulista, Campus Araraquara, Departamento de Fármacos e Medicamentos, Araraquara 14800-903 SP, Brazil; jebernegossi@hotmail.com; 2 Faculdade de Ciências Farmacêuticas, UNESP—Univ. Estadual Paulista, Campus Araraquara, Departamento de Análises Clínicas, Araraquara 14800-903 SP, Brazil; lfmarise@gmail.com

**Keywords:** photodynamic therapy, cancer, nanotechnology, drug delivery systems

## Abstract

Photodynamic therapy (PDT) is a promising alternative approach for improved cancer treatment. In PDT, a photosensitizer (PS) is administered that can be activated by light of a specific wavelength, which causes selective damage to the tumor and its surrounding vasculature. The success of PDT is limited by the difficulty in administering photosensitizers (PSs) with low water solubility, which compromises the clinical use of several molecules. Incorporation of PSs in nanostructured drug delivery systems, such as polymeric nanoparticles (PNPs), solid lipid nanoparticles (SLNs), nanostructured lipid carriers (NLCs), gold nanoparticles (AuNPs), hydrogels, liposomes, liquid crystals, dendrimers, and cyclodextrin is a potential strategy to overcome this difficulty. Additionally, nanotechnology-based drug delivery systems may improve the transcytosis of a PS across epithelial and endothelial barriers and afford the simultaneous co-delivery of two or more drugs. Based on this, the application of nanotechnology in medicine may offer numerous exciting possibilities in cancer treatment and improve the efficacy of available therapeutics. Therefore, the aim of this paper is to review nanotechnology-based drug delivery systems for photodynamic therapy of cancer.

## 1. Introduction

The combination of dyes and light for treatment of diseases dates back to ancient Egypt, where conditions such as psoriasis and vitiligo were treated with vegetal-derived substances and sunlight [1]. However, it was only in the beginning of 20th century that modern PDT (photodynamic therapy) began with the discoveries of O. Raab [2] and H. von Tappeiner and A. Jesionek [3]. O. Raab observed the deleterious effect of treating *Paramecium caudatum* with acridine orange and accidentally exposing it to sunlight [2]. In 1903, von Tappeiner and Jesionek reported the tumoricidal effect of eosin associated with exposure to the white light on skin tumors. They conceived the term “photodynamic action” [3].

PDT is considered a promising alternative treatment modality to current treatments against several types of cancers. A non-toxic dye (capable of transferring light energy to other molecules, *i.e.*, a photosensitizing agent) is administered to the patients with subsequent exposure to a light source of specific wavelength that leads to the death of the target cell via oxidative damage [4].

**Figure 1:**
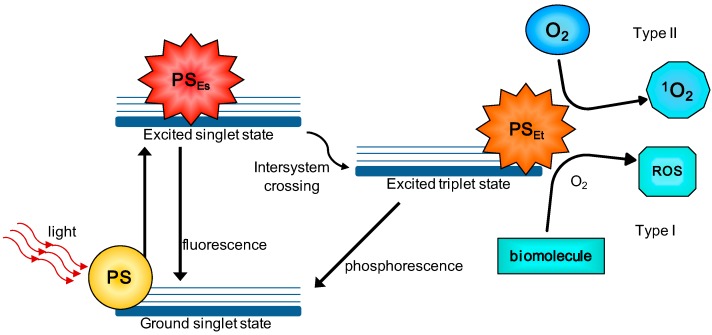
Type I and Type II reactions in PDT (photodynamic therapy). Schematic Jablonski’s diagram showing PDT’s mechanism of action. Following light absorption, the PS reaches an excited singlet state. After an intersystem crossing, the PS, now in a triplet excited state, can react in two ways: react with biomolecules through a hydrogen atom (electron) transfer to form radicals, which react with molecular oxygen to generate ROS (type I reaction); or, the PS in its triplet state can react directly with oxygen through energy transfer, generating singlet oxygen (Type II reaction). PS: photosensitizer; PS_Es_: PS excited singlet state; PS_Et_: PS excited triplet state; ROS: reactive oxygen species; ^1^O_2_: singlet oxygen.

Three components act simultaneously in PDT: a photosensitizer (PS), a light source, and oxygen. The PS and light source must be harmless to the target cell [5]. When the PS is excited by light of a specific wavelength, its interactions with the surroundings can follow two pathways. These pathways are named Type I and Type II reactions, as illustrated in Figure 1. In type I reactions, the PS in its excited triplet state reacts with biomolecules (*i.e.*, lipids, proteins, and nucleic acids), transferring hydrogen atoms via the radical mechanism. It generates free radicals and radical ions (radical type depends on the target molecule, *i.e.*, lipids, proteins, or nucleic acids) that then react with oxygen resulting in reactive oxygen species (ROS) generation [6,7,8,9].

Type II reactions are based on a phenomenon called triplet–triplet annihilation. In these reactions, the PS in its excited triplet state reacts with oxygen in its triplet ground state. This yields singlet oxygen that is highly reactive and cytotoxic. Both types of reactions take place at the same time. The balance between these two processes depends on the PS being used, the concentrations of oxygen and substrate, and affinity of the PS with the substrate [6,7,8,9]. However, singlet oxygen generation pathway *i.e.*, Type II reactions are considered the principal mechanism of [10].

ROS and singlet oxygen have high reactivity and short half-life. Due to this, PDT directly affects only those biological substrates that are close to the region where these species are generated, usually within a 20 nanometers radius [11]. Therefore, PS localization is a primary factor in drug release studies to target tissues because it selectivity promotes localized sensitization.

## 2. Clinical PDT against Cancer

Although the discovery of photodynamic effect was based in the field of microbiology, the use of PDT against microbial infections was severely cutback after the introduction of antibiotics in the 1940s. However, it is being revisited in the last few decades as an alternative therapy due to the increasing bacterial resistance to existing antibiotics [6]. PDT advancements occurred primarily for the treatment of cancer and non-infectious diseases, such as vitiligo, macular degeneration, and psoriasis [12]. Various clinical protocols have been approved for the treatment of pre-malignant and malignant lesions since its introduction in clinical practice in the 1980s [7,8]. The cancer types treated using PDT include cancers of the digestive tract [13,14] and esophagus [15,16], lesions of the head and neck [17], lung and malignant pleural mesothelioma [18], cervix, bladder, and non-melanoma skin cancer, and basocellular carcinoma [8], among others.

Clinical PDT protocol usually includes a standard dose of the drug, a dose from a light source, and a drug-light interval. However, the clinical outcomes may vary enormously. This is probably due to poor light delivery to the target tissue, resistance to the drugs (e.g., in tumors), or metabolism of the PS in individual patients [19].

Light delivery can be enhanced by using deeper wavelengths (from 600 to 800 nm, the “therapeutic window”) [20,21] or a different illumination approach, such as interstitial instead of superficial light delivery [22]. PDT-resistant tumor cells were isolated *in vitro*. The mechanisms of resistance to PS are similar to those described for general drugs, including increased drug inactivation, decreased uptake, drug efflux, and altered intracellular trafficking [23]. The combination of at least two different treatment modalities is one of the strategies to overcome tumor resistance. For example, combination of PDT and chemotherapy was extremely efficient in eliminating cancer cells as shown by our group [21] and others [24,25,26].

PDT has several advantages over conventional cancer treatment approaches. First generation PSs cause minor self-limited photosensitivity to the eyes and skin; however, PDT has no long-term side effects when properly used [27]. It is less invasive than surgical procedures and the side effects usually last for a shorter period than chemotherapy or radiotherapy. Furthermore, PDT procedures are most often performed on an outpatient basis. Moreover, it also destroys the vasculature associated with the tumor besides the tumor itself, which contributes greatly to tumor death [11]. Furthermore, PDT can be directed to a target tissue very precisely due to its dual selectivity (only the irradiated area receives the PS and the light simultaneously). It can be repeated several times at one location if necessary, unlike radiation. There is little or no scarring after healing. Lastly, it generally costs less than other cancer treatments [28].

However, like every therapy, PDT has some limitations. The photodynamic effect takes place selectively at the irradiated site, which makes it impossible for PDT to be used in disseminated metastases with currently available technology, and this does not allow for a whole body irradiation with the appropriate light intensity [28]. Tissue and tumor oxygenation is crucial for the photodynamic effect to take place; therefore, tumors surrounded by necrotic tissue or dense tumor masses lead to impaired PDT. Finally, the likelihood of the precise light delivery to the target tissues is the most important point when considering PDT as a treatment option. Therefore, deep tumors (not easily accessible without surgical intervention) are hard to treat due to low tissue penetration of visible light [29]. The advantages and disadvantages of PDT are summarized in Table 1.

### 2.1. Photosensitizers

The choice of PS is critical for successful treatment with PDT. The PS should be preferably excited by light of a wavelength in the therapeutic window (600–700 nm), which has greater capacity for tissue penetration. It must be non-toxic to the cells in the dark (*i.e.*, it should not cause cell death in the absence of light). Furthermore, it must be selectively captured and/or retained by the target cells. Lastly, it should be able to induce immunogenic cell death (characterized by changes in the composition of the cell surface to release soluble mediators that activate dendritic and T cells) that promotes the development of a specific immune response against malignant tumors [4,7,30]. In this section, a few photosensitizers that generally meet the above-mentioned principles have been described. Furthermore, some *in vitro* and/or *in vivo* applications are also described.

#### 2.1.1. Methylene Blue (Phenothiazine Derivative)

Methylene blue (MB) is an organic dye with both fluorescent and photosensitizing properties [31,32,33]. This PS effectively inactivates virus and bacteria (*in vitro*), and kills malignant cells (*in vivo*) [34,35,36]. The positive charge and its low molecular weight promote an enhanced interaction with bacteria and mammalian cells. This makes it a suitable option for PDT of infections and cancer [36,37].

Hydrophilic/lipophilic balance and net positive charge on MB allows it to easily penetrate biological membrane [36]. It is most commonly used as a PS in antimicrobial PDT, for the treatment of *Candida albicans*, *Enterococcus faecalis*, and *Escherichia coli* infections. MB application as a PS in anticancer PDT is increasing. It has shown promising results as evidenced by several studies including from our group [38], Tardivo and colleagues [36], and Wagner and colleagues [39], among others. MB application in anticancer PDT is especially interesting because of its lower cost and easier availability compared to conventional PSs. Samy *et al.* [40] used MB–PDT to treat basal cell carcinoma in 17 patients. Out of the 17 patients, 11 achieved a complete response with good cosmetic outcome and minimal side effects. Currently, MB–PDT is limited to clinical studies, with no approved clinical protocol.

Although MB has substantial water solubility, its solutions are rapidly eliminated after intravenous administration due to reduction into leucoMB in erythrocytes and peripheral tissues [41]. Since leucoMB has no photosensitizing action, protecting MB from blood and peripheral reduction is essential when considering IV administration. Nanoparticles prepared from PGLA and chitosan, two biocompatible polymers, can be used to improve MB stability.

#### 2.1.2. Photogem^®^ (Hematoporphyrin Derivative, HpD)

Photogem^®^ is a hematoporphyrin derivative from Russia (Moscow). It is a first generation PS produced from animal blood. Its chemical, photophysical, diagnostic, and therapeutic characteristics are identical to Photofrin^®^. It has been approved for human use by the Pharmacology State Committee of the Russian Federation and by the Brazilian Health Surveillance Agency (ANVISA). It consists of a mixture of monomers and oligomers with an absorption spectrum between 500 and 630 nm [42].

Photogem^®^ is usually administered systemically and its main adverse reaction is photosensitivity for a few weeks after administration. The adverse reaction can be limited by incorporating Photogem^®^ into drug delivery systems or targeting systems, which allows for a slow release of the PS to the body or its specific delivery to the tumor. However, it has low light absorption within the therapeutic window (from 600 to 700 nm) and induces prolonged photosensitivity. Therefore, second generation photosensitizers with better absorption features and fewer adverse reactions were developed, including 5-aminolevulinic acid (ALA, Levulan^®^) and chlorins [9].

#### 2.1.3. Chlorins

Chlorins and bacteriochlorins have a strong light absorption between 640 and 700 nm and are found in natural products. For example, chlorins are found in chlorophyll-a (present in some species of *Spirulina*) and bacteriochlorins are found in *Rhodobacter capsulatus* bacteria [43]. Chlorins are hydrophilic reduced porphyrins; therefore, their basic structure is similar to porphyrins [44,45]. Several chlorin derivatives have been studied for use as PSs, including mono-l-aspartyl chlorin e6 (NPe6). It has two important properties: a high quantum yield of singlet oxygen (0.70) and an intense light absorption in wavelengths within 650 and 680 nm (therapeutic window) [46]. Some synthetic chlorins, such as meso-tetrakis (m-hydroxyphenyl) chlorin (m-THPC) and the benzoporphyrin derivative BPDMA, show promising biological activity [47].

Photodithazine^®^ (PDZ) is another PS drug (also from Russia) from the chlorins family. It is obtained from cyanobacteria *Spirulina platensis* and its structure is modified by the addition of 0.5% *N*-methyl-d-glucosamine as a solubilizing and stabilizing agent [48]. It is a second-generation PS which induces little or no skin photosensitivity [49].

Chlorins have been extensively investigated for their potential to treat oral cancer [50,51]. For example, Parihar *et al.* [50] studied the potential of chlorin-PDT to treat oral squamous cell carcinoma, both *in vitro* and *in vivo*. They reported extensive cellular damage and complete tumor regression within a week after chlorin-PDT.

Although chlorins exhibit good water solubility and stability, aqueous solutions are not always the preferred delivery system to administer a PS for treating lesions of the oral cavity. Incorporation of the chlorins into a mucoadhesive delivery system is a better choice in such cases. It increases the residence time of the dosage form at the site of absorption and improves the overall outcome [52]. For example, chlorin e6 (the most commonly used chlorin) has been incorporated into a variety of nano-vehicles prepared from chitosan, human serum albumin, silica, iron oxide, hyaluronic acid, and various polymeric nanoparticles [53].

#### 2.1.4. Curcumin

Curcumin is a polyphenolic compound isolated from *Curcuma longa* L. that has been used for centuries as a medicine, dye, and spice. It can also be used as a PS. Curcumin has a variety of pharmaceutical applications in wound treatment, liver diseases, blood purification, joint inflammation, and for antimicrobial effects [54].

Curcumin has a wide light absorption range from 300 to 500 nm (maximum peak at 430 nm) and exerts its biological activity in micromolar concentrations [55]. It presents a strong potential as a PS for the treatment of localized superficial infections caused by either fungi or bacteria [56] and for the treatment of superficial tumors, especially skin and oral cavity cancers [57]. Curcumin is more efficacious to treat “superficial” infections because it absorbs blue light, which has poorer tissue penetration than red light (therapeutic window), which is not suitable for deeper lesions [58]. The main limitation of using curcumin in PDT is its poor water solubility (<0.1 mg/mL), which leads to poor bioavailability and suboptimal pharmacokinetics. This limitation can be overcome by using nanotechnology-based drug delivery systems to improve curcumin solubility. For instance, Yallapu *et al.* [59] designed curcumin-loaded cellulose nanoparticles to treat prostate cancer, *in vitro*. They demonstrated that curcumin-loaded cellulose nanoparticles showed improved efficacy compared to free curcumin.

#### 2.1.5. Phthalocyanines

Phthalocyanines are second-generation PSs similar to porphyrins. They have superior photophysical and photochemical properties [60], including: simple synthesis and possibility of modification to alter hydrophilicity; high photo- and chemical-stability; long-wavelength absorption with high extinction coefficients (much more intense absorption in the 650–750 nm region); and high singlet oxygen quantum yields [61].

Like porphyrins, phthalocyanines coordinate metal ions within their core, which offers numerous options to control their physical properties by synthetic modifications. However, phthalocyanines lack tumor cell specificity [62]. To address this drawback, phthalocyanines have been conjugated to tumor-targeting peptides [61] or incorporated in liposomes [62]. For example, Muehlmann *et al.* [63] conjugated aluminum-phthalocyanine chloride (AlPc, a highly hydrophobic phthalocyanine derivative) with poly (methyl vinyl ether-co-maleic anhydride) nanoparticles, which increased the singlet oxygen generation capacity of AlPc by 10-fold compared to its free form, thus significantly improving its photodynamic activity.

Furthermore, systems combining a PS and metal nanoparticles (hybrid nanostructures) are also highly attractive for cancer treatment due to the extended PDT capabilities [64]. The hybrid nanostructures containing phthalocyanines and metal nanoparticles enhance the therapeutic effects by: increasing the singlet oxygen quantum yield; the synergetic effect by means of metal nanostructures and singlet oxygen generated by phthalocyanine; and facilitating in phthalocyanine cellular uptake [65].

#### 2.1.6. Hypericin

Hypericin (HYP) is a naturally occurring red plant pigment extracted from *Hypericum perforatum* L. (St John’s Wort) [66,67], which has been used for centuries in traditional medicine. Extensive biochemical research in the last three decades showed that HYP is a multifunctional agent and has medicinal applications, such as antidepressant, antineoplastic, antitumor, and antiviral (human immunodeficiency and hepatitis C virus) agent [68]. It has drawn increased interest as a PS due to its photochemical properties, such as high quantum yields of singlet oxygen, light absorption close to the therapeutic window (590 nm), tumor selectivity, and low production costs. Additionally, it shows low photobleaching, low cytotoxicity in the absence of light, and no mutagenicity. Several *in vitro* and *in vivo* studies have shown its anticancer potential in PDT [67].

For example, Kleeman *et al.* [66] showed that HYP-PDT is effective in eliminating melanoma cells *in vitro*. Similarly, Barathan *et al.* [69] found that HYP-PDT could eliminate hepatocellular carcinoma cells. Furthermore, antitumor properties of HYP-PDT have also been demonstrated *in vivo* and in clinical studies. These studies highlighted the potential of HYP-PDT to treat recurrent mesothelioma, basal-, and squamous-cell carcinoma [68,70,71,72]. However, clinical applications of HYP-PDT for other types of cancer are still lacking.

HYP is highly hydrophobic. It is soluble in alkaline aqueous solutions, organic bases, polar organic substances, and biological media. However, it is insoluble in water and non-polar solvents [67], which means it readily aggregates in water. This impairs the administration of the PS (HYP) *in vivo*, and leads to a drastic loss of its photodynamic activity [63,73,74].

Lima *et al.* [75] demonstrated that nanotechnology has an important role in photodynamic therapy. They entrapped HYP in solid lipid nanoparticles. It increased cell uptake of the PS by 30% and improved the photodynamic effect.

**Figure 2:**
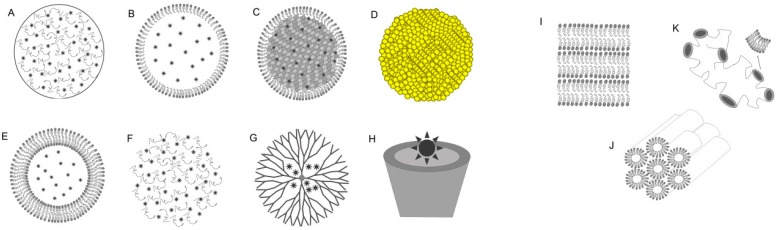
Schematic representation of nanotechnology-based drug delivery systems such as (**A**) polymeric nanoparticles (PNPs); (**B**) nanostructured lipid carriers (NLCs); (**C**) solid lipid nanoparticles (SLNs); (**D**) gold nanoparticles (AuNPs); (**E**) liposomes; (**F**) hydrogels; (**G**) dendrimers; (**H**) cyclodextrin; and (**I**) lamellar; (**J**) hexagonal; or (**K**) cubic mesophases liquid crystallines for photodynamic therapy of cancer.

Therefore, nanotechnology-based PS delivery represents an emerging approach to improve the outcome of cancer PDT. Development of the nanotechnology-based drug delivery systems, such as illustrated in the Figure 2, can facilitate precise PS intracellular delivery.

Additionally, nanotechnology-based drug delivery systems have advantages such as: (1) improved delivery of poorly water-soluble PS; (2) facilitating transcytosis of PS across tight epithelial and endothelial barriers; (3) delivery of large macromolecular PS to intracellular sites of action; and (4) co-delivery of two or more drugs (including chemotherapeutics) for combination therapy [76].

The following section presents the efforts of researchers to create novel strategies to develop nanotechnology-based drug delivery systems in cancer–PDT.

## 3. Nanotechnology-Based Drug Delivery Systems

### 3.1. Nanoparticle Systems

Nanoparticles (NPs) are sub-micrometer size particles. They have numerous advantages as a PS delivery system, such as (i) protection of the PS against enzymatic degradation; (ii) the control of PS release allowing a constant and uniform concentration into target cells; (iii) the ability to penetrate target cells due to their submicron size; (iv) biocompatibility and resorbability through natural pathways; and (v) photostability [77].

NPs can be classified as polymeric nanoparticles (PNPs), solid lipid nanoparticles (SLNs), nanostructured lipid carriers (NLCs), and metallic nanoparticles (MNs), depending on the material of which they are made [78].

PNPs are prepared from natural or synthetic polymers such as PLGA (poly-d,l-lactide-co-glycolide), PLA (polylactic acid), PCL (poly-caprolactone), PAC (Poly-alkyl-cyano-acrylates), chitosan, and gelatin, among others [79,80]. Ohulchanskyy *et al.* [80] developed PNPs for the treatment of colon cancer. They synthesized ultralow size organically modified silica nanoparticles (SiNPs) that were covalently linked with iodobenzylpyropheophorbide PS by the alkaline hydrolysis and polycondensation of the organotrialkoxysilane precursors within the nonpolar core of Tween-80/water micro-emulsion. They showed that the prepared SiNPs had a great affinity for cancer cells, evidencing their great potential for cancer treatment and diagnosis by PDT.

Dioctyl sodium sulfosuccinate and sodium alginate PNPs were fabricated using a multiple-emulsion cross-linking process by Khdair *et al.* [81]. They investigated the ability of these PNPs to enhance the therapeutic efficacy of the PS MB in two cancer cell lines, MCF-7 and 4T1. NP-encapsulated MB enhanced the accumulation of MB in the nucleus and increased the ROS production, which improved its anticancer photodynamic efficacy *in vitro.* They suggested that this NP system proved to be a promising MB delivery system for anticancer PDT.

Similarly, Yu *et al.* [82] developed Photosan PS-loaded NPs from a amphiphilic sodium alginate derivative conjugated with cholesteryl residues using a simple self-assembly method in an aqueous solution. They showed that the PNPs improved the phototoxicity of Photosan against human pancreatic cancer cells and induced the cell death by apoptosis.

Yuan and Liu fabricated paclitaxel-loaded PNPs based on a poly (ethylene glycol) (PEG)-grafted conjugated polyelectrolyte (CPE), which itself serves as a photosensitizer. This strategy to combine both chemo and photodynamic cancer therapy against U87-MG and MCF-7 cells resulted in higher toxicity than each treatment alone. It showed that this novel PNP system offers a potential alternative for the cancer treatment [83]. Jin *et al.* [84] developed hypocrellin A (HA) PS-loaded blue-emitting upconversion nanoparticles (UCNPs). The assessment of anticancer activity of HA-loaded NPs against human epithelial lung cancer cells (A549) and human cervical cancer cells (HeLa) showed an efficient cancer cell killing ability.

Photonic explorers for biomedical use by biologically localized embedding (PEBBLE) is an interesting technology for development of NPs. El-Daly *et al.* [85] developed NPs based on PEBBLE using organically modified silicate (ormosil) as the matrix to entrap indocyanine green (ICG) PS. They investigated the photodynamic effect of ICG-loaded NPs and free ICG on human breast adenocarcinoma cells (MCF-7) and hepatocellular carcinoma cells (HepG2). They found that the NPs increased the stability of ICG while maintaining the ICG phototoxic power, and suggested that PDT with ICG-loaded NPs causes lesser oxidative damage to DNA than in PDT with free ICG.

In the early 1990s, SLNs made from solid lipids and particles were introduced as an alternative to the PNP. SLNs do not present the disadvantages that some PNPs present, such as cytotoxicity and difficult large-scale manufacturing. Moreover, the solid lipids are better tolerated and SLNs are cost-effective than PNPs [86]. However, the SLNs also have some limitations, such as low encapsulation efficiency, drug expulsion during storage, and crystallization. Therefore, NLCs were developed to overcome these problems. NLCs are formed by solid lipid and liquid lipid phases, which creates a disorganized matrix, preventing the solid lipid crystallization and increasing the drug payload in the NLC. Both SLNs and NLCs can be prepared by hot or cold high-pressure homogenization, by microemulsion or by the precipitation method [86].

Youssef *et al.* [87] prepared and physicochemically characterized SLNs using the microemulsion-based technique to deliver HYP. HYP-loaded SLNs showed good compatibility between HYP and lipids (forming the cores of SLN). Moreover, HYP encapsulation in SLNs improved its photostability. Interestingly, phototoxicity of HYP was decreased by encapsulation in SLNs, which might be due to the quenching-induced deactivation of HYP resulting from the compactness and thickness of the SLN structure. Using the ultrasonication technique, Lima *et al.* [75] prepared HYP-SLNs with high entrapment efficiency and drug loading capacity. In contrast to Youssef *et al.* [87], they showed that HYP-loaded SLNs increased the cell uptake by 30% and improved the cytotoxicity by 26%. This improvement was most likely due to the smaller size of SLNs (153 nm) in this study and the preparation technique that led to an increase in cellular internalization mediated by the SLN vehicle and increased the concentration of HYP inside the cells [75].

Another type of NP that excelled greatly as a drug delivery system is the metallic nanoparticles (MNs). MNs are NPs functionalized mainly by gold (AuNPs). Due to monolayer tunability, the AuNPs can be loaded with the drug by different mechanisms, such as by covalent and non-covalent conjugation. Furthermore, it is easy to control the target, stability, and release of the drug from AuNPs. There are several ways to synthesize these NPs with varying core sizes. For example, the reduction of AuCl (PPh3) with diborane of sodium borohydride is usually used to prepare AuNPs with up to 2 mm core size. NPs from 1.5 nm to 6 nm core sizes can be fabricated by biphasic reduction of thiol capping agents. Similarly, NPs with up to 150 nm diameter can be formed by citrate reduction of gold salts or ripening approaches [88]. Shi *et al.* [89] prepared and PEGylated iron oxide NPs (IONPs) decorated onto the surface of fullerene (C60) obtaining a multifunctional C60-IONP-PEG nanocomposite. Subsequently, they conjugated hematoporphyrin-monomethyl-ether (HMME) PS to the C60-IONP-PEG and tested it against B16-F10 cells *in vitro* and in an *in vivo* murine tumor model. The results demonstrated that C60-IONP-PEG/HMME markedly enhanced the photodynamic cancer cell killing effect compared to free HMME.

#### 3.1.1. Liposomes

Liposomes are unilamellar or multilamellar nanometer scale systems spontaneously formed when phospholipids are dispersed in an aqueous medium. Clinically, they are the most established controlled drug delivery system due to their structural flexibility and their ability to incorporate a variety of hydrophilic and hydrophobic drugs, besides being biocompatible and biodegradable. Therefore, liposomes can be used to incorporate lipophilic and hydrophilic PSs used in cancer PDT [90,91].

For instance, Bovis *et al.* [92] investigated the efficacy of the clinical PDT PS m-THPC incorporated in liposomes following intravenous administration to normal and tumor-bearing rats. Liposomes were prepared as a 9:1 mixture of dipalmitoylphosphatidylcholine and dipalmitoylphosphatidylglycerol with 2% and 8% (molar equivalent ratio) pegylation using 1,2-distearoyl-sn-glycero-3-phosphoethanolamine-*N*-[amino(polyethylene glycol)-2000]. The results showed that m-THPC-loaded liposomes improved tumor selectivity in comparison to the commercial formulation. It was attributed to the higher tumor uptake and blood plasma concentrations, which was suggested to reduce the damage to healthy cells. Furthermore, it was also suggested that the cost of treatment could be reduced by using liposomes because a lower dosage of m-THPC was required when loaded in liposomes.

Derycke *et al.* [93] evaluated the potential of aluminum phthalocyanine tetrasulfonate (AlPcS4)-loaded liposomes as an effective and safe strategy for PDT of superficial bladder cancer. They incorporated AlPcS4 in unconjugated liposomes (Lip-AlPcS4) or transferrin-conjugated liposomes (Tf-Lip-AlPcS4) and observed the accumulation of free AlPcS4, Lip-AlPcS4, and Tf-Lip-AlPcS4 in human AY-27 transitional-cell carcinoma cells and in an orthotopic rat bladder tumor model by fluorescence microscopy. They found that Tf-Lip-AlPcS4 accumulated substantially in AY-27 cells. Furthermore, *in vivo* results showed that Tf-Lip-AlPcS4 selectively accumulated in tumors. Based on this, this formulation can be a promising vehicle to selectively deliver PSs to bladder tumor cells.

The efficacy of the PDT treatment performed by an intravenous injection of benzoporphyrin derivative monoacid ring A (BPD-MA)-entrapped liposomes and polycation liposomes (PCLs) followed by exposure to a laser light (689 nm with 150 J/cm^2^ fluence 15 min post injection) was investigated by Takeuchi *et al.* [94]. They evaluated the efficiency of uptake in tumor-derived angiogenic vascular endothelial cells. The results showed that BPD–MA entrapped in PCLs induced a higher destruction of angiogenic vessels and subsequent tumor suppression by vessel occlusion than the other liposome type. This was probably due to the strong electrostatic adhesion between the polycation and the plasma membrane, improving the phototherapeutic efficacy.

Pierre *et al.* [95] developed a novel liposome formulation composed of mammalian stratum corneum lipids (stratum corneum lipid liposomes; SCLLs) to study 5-aminolevulinic acid (5-ALA) skin delivery intended for skin cancer PDT. SCLLs improved the delivery of 5-ALA into viable epidermis and dermis compared to 5-ALA aqueous solution. The authors suggested that the similarity in structure of this liposome and a human cell could have favored the interaction of SCLLs with the skin. Therefore, SCLLs can be a suitable system for the topical delivery of 5-ALA.

Nombona evaluated the photodynamic efficiency of 1,6-hexanedithiol tetra-substituted zinc phthalocyanine PS-loaded liposomes or gold nanoparticles on MCF-7 cells. The results demonstrated that PS-loaded liposomes caused more damage to the breast cancer cells than gold nanoparticles. It again suggests that liposomes can be a potential formulation for cancer PDT [96].

Barbugli *et al.* [97] studied the uptake of chloroaluminium phthalocyanine (ClAlPc) liposomes in metastatic melanoma cells as well as its PDT efficiency in 3D cell culture models using metastatic melanoma cells WM1617 (s1–s13). They found that the ClAlPc molecule was taken up and present in the cytoplasm. Furthermore, it inhibited the growth of spheroids and colonies in 3D cell cultures, suggesting that this liposome efficiently targets the melanoma cells.

#### 3.1.2. Hydrogels

Hydrogels are systems comprised of polymeric materials that are capable of absorbing water and present a three-dimensional mesh structure [98].

These systems are widely used for the controlled release of hydrophilic drugs [99], mainly due to the ease of dispersing the drug in the matrix. Additionally, they are biocompatible and have physical properties that are similar with living tissues [100]. In addition, hydrogels can possibly be used for applications delivery of hydrophilic PSs for PDT, including PAD-S31 (13,17-bis(1-carboxypropion)carbamoylethyl-3-ethenyl-8-ethoxyiminoethylidene-7-hydroxy-2,7,12,18-tetramethyl-porphyrin sodium), Mono-l-aspartyl chlorin, FA-PEG-PheoA, and aluminum (III) phthalocyanine chloride tetrasulfonic acid (Al-4), among others [101,102,103].

For example, Saboktakin *et al.* [104] investigated biodegradable NP hydrogels loaded with the PS temoporfin (mTHPP) for cancer-PDT. Chitosan hydrogels were prepared from lyophilized conjugates of chitosan standards (stored at −80 °C). The mTHPP-loaded nanoparticles were prepared from 0.2% chitosan hydrogel plus 0.04% mTHPP dispersed in deionized water by stirring. After approximately 3 h, the sample was sprayed into a liquid nitrogen bath cooled down to 77 K. The formed frozen droplets which were dried by sublimation. The NP analysis showed that mTHPP has interesting features, which remained stable during the analyzed period. They concluded that these NPs are suitable as carriers for PDT.

Recently, porphyrin-loaded hydrogels were studied for controlled chemotherapeutics release and PDT. Poly-(ε-caprolactone)-b-poly-(ethylene glycol) (SPPCL-b-PEG) copolymer was synthesized and used as a building block for constructing supramolecular hydrogels. The hydrogel was prepared by mixing an aqueous solution of amphiphilic SPPCL-b-PEG copolymer and α-CD at 37 °C. The authors found that the new hydrogel system was more stable and showed significantly improved sustained release compared to copolymer micelles. Rheologic characterization showed that it was a reversible thixotropic system. Moreover, they also found that hydrogels exhibited efficient singlet oxygen generation upon irradiation by visible light (650 nm), proving to have the potential for the treatment of cancer [105].

#### 3.1.3. Liquid Crystalline Systems

Lyotropic liquid crystal systems have attributes of both liquids (because they are fluids) and solids (as they present an organized structure). They are formed by surfactants, more precisely by their solvates or hydrates [106]. They are briefly characterized as mesophases lamellar, hexagonal or cubic, and have been extensively investigated by researchers [107] because they are non-toxic, biodegradable, and have bioadhesive properties which also contribute to their applications in drug delivery of PSs [108]. The lamellar liquid crystals exhibit low interfacial tensions with oil despite their minimal oil solubilization and they have low viscosities [109]. They are formed by parallel layers of surfactant bilayers separated by layers of solvent [106]. Hexagonal mesophase can be visualized under polarized light microscopy as long cylinders arranged in a two-dimensional array [99]. Finally, the cubic mesophase presents further complicated structures to be visualized, usually presenting a cubic symmetry. Additionally, the tetragonal and rhombohedral phases were also detected in some systems [110].

Researchers recently investigated nanoparticles of lyotropic liquid crystals loaded with a chlorine derivative, a PS used for PDT of skin cancer. They prepared the hexagonal phase nanodispersion by mixing monoolein, oleic acid, poloxamer, and water at a temperature of 45 °C. They found that the particle of size was 161 ± 4 nm and the polydispersity index was 0.175 ± 0.027, and that the hexagonal liquid crystalline phase remained stable even after the drug addition. Based on the penetration studies, they suggested that the absorption of the PS loaded in the hexagonal system was significantly higher than in the control (PS in polyethylene glycol). They showed that the PS incorporated in the hexagonal liquid crystalline phase nanodispersion had deeper distribution among the skin layers when compared to the control. Finally, they concluded that the nanodispersion showed potential for the delivery of the PS into the skin, which is the crucial condition for successful topical PDT [111,112].

#### 3.1.4. Dendrimers

Dendrimers are highly branched polymers and have a very precisely defined diameter, usually about 1–10 nm. Their main advantage is the predictability and control of size and number of functional groups available for modifications. This provides greater certainty in predicting the amount of incorporated drug. Therefore, it enables reproducible pharmacokinetics, which makes dendrimers an interesting drug delivery system for PDT [113].

There are three ways to conjugate PS in dendrimers: (i) PS is trapped in the voids of a dendrimer; (ii) PS is covalently bound to the dendrimer; (iii) PS is used as a scaffold to form a dendrimer [114].

The second approach to conjugate PS with a dendrimer was used by Narsireddy *et al.* [115] who conjugated 5,10,15,20-tetrakis(4-hydroxyphenyl)-21*H*,23*H*-porphine (PS) and nitrilotriacetic acid (NTA) group in a dendrimer. They conjugated a peptide (specific to human epidermal growth factor 2) to the NTA group on the dendrimer to act as a tumor-targeting peptide for targeted *in vitro* and *in vivo* PDT. They found that PS-dendrimers were efficient in PDT-mediated cell death assays in human epidermal growth factor receptor 2 (HER2) positive SKOV-3 cells, and PS-dendrimers significantly suppressed the tumors in a xenograft animal tumor model.

Similarly, Rodriguez *et al.* [116] reported that the conjugation of ALA dendrimers enhances porphyrin synthesis. Therefore, they evaluated the ability of ALA dendrimers carrying 6 and 9 ALA residues (6m-ALA and 9m-ALA) to photosensitize carcinoma cells. They found that at low concentrations porphyrin synthesis from dendrimers was higher than from ALA. However, porphyrin synthesis was similar from both compounds at high concentrations. Furthermore, skin application of ALA dendrimers showed no diffusion to distant skin sites, indicating a promising use of the ALA macromolecules in superficial cancer models.

Kojima *et al.* [117] synthesized poly (ethylene glycol) (PEG)-attached dendrimers derived from poly (amido amine) (PAMAM) and poly (propylene imine) (PPI) dendrimers (PEG-PAMAM and PEG-PPI) using rose bengal (RB) and protoporphyrin IX (PpIX) as PSs. They showed that PEG-PPI kept the PSs more stable than PEG-PAMAM due to its inner hydrophobicity. Moreover, PpIX loaded-PEG-PPI exhibited more cytotoxicity, which may have been caused by the high level of singlet oxygen production and the efficient delivery to mitochondria. Hence, it can be suggested that this dendrimer is a promising vehicle for PDT.

Nishiyama *et al.* [118] showed that porphyrin-loaded dendrimers with ionic peripheral groups (DPs) spontaneously generated polyion complex (PIC) micelles through electrostatic interactions with oppositely charged block copolymers. Moreover, there was no self-quenching of the dye molecule inside the micellar core due to a unique DP structure that lead to a remarkable *in vitro* photocytotoxicity. This dendrimer also showed potential in treatment of choroidal neovascularization (CNV) in rats without any sign of side effects.

Karthikeyan *et al.* [119] investigated the potential of Rose Bengal (RB)-loaded polyamidoamine dendrimers as well as their phototoxic efficiency on Dalton’s Lymphoma Ascite (DLA) cancer cell lines. They showed that this dendrimer acted as a suitable PS drug delivery system releasing above 80% of RB in 72 h. Furthermore, RB-loaded dendrimer exhibited minimized dark toxicity and enhanced phototoxicity to DLA cells compared to the dark phototoxicity of free RB; therefore, dendrimers can be a promising drug delivery system for PDT.

#### 3.1.5. Cyclodextrin

Natural cyclodextrins (CDs) are cyclic oligosaccharides obtained by action of cyclodextrin-α-glycosyl transferase enzyme (CGTase). They have six, seven, or eight glucose units connected by α-1,4 bonds named, respectively, α-, β-, and γ-CD [120]. Several modified CDs have been synthesized in order to improve the structures of natural CDs, making them suitable for pharmaceutical applications. They have a shape similar to a cone, therefore, a nonpolar internal cavity is formed, allowing the formation of inclusion complexes of lipophilic drugs [121].

Making use of this, Conte *et al.* [122] developed biodegradable nanoassemblies based on heptakis (2-oligo(ethyleneoxide)-6-hexadecylthio-)-β-CD (SC16OH) and zinc-phthalocyanine (ZnPc) to overcome poor aqueous solubility of phthalocyanines. They found potential photodynamic anticancer effects in HeLa cancer cells, suggesting a promising Zn-Pc delivery system.

Similarly, Lourenço *et al.* [123] prepared phthalocyanines conjugated with α-, β-, and γ-CDs. They found that both Pc-α-CD and Pc-γ-CD showed a potential photoactivity against UM-UC-3 human bladder cancer cells.

## 4. New Approaches and Challenges

The greatest challenge in oncology is selectively killing the tumor cells without harming healthy cells. In this regard, PDT offers clear advantages over other therapeutic modalities, such as chemotherapy and radiotherapy. It requires a concomitant presence of PS and light for the photodynamic effect to take place. Therefore, the photodynamic action can be preferentially localized in malignant tissues resulting in irreversible destruction of tumor cells. Nonetheless, the systemic administration of PS can result in photosensitivity of skin and eyes, forcing the patient to avoid direct light exposure for a period of up to 30 days after each administration. Furthermore, there is a major difficulty in establishing an appropriate interval between PS administration and light irradiation (drug–light interval), which is crucial for the success of PDT. If light irradiation is too early, it will only affect the tumor vasculature. Accordingly, if the light irradiation is too late, it will have a diminished effect due to PS clearance [8]. Therefore, these challenges motivate researchers to improve PDT using nanotechnology-based drug delivery systems.

These NP drug delivery systems offer benefits for PSs, such as: targeting only tumor tissues; enhancing its permeability and retention (EPR effect); and increasing its retention time in the malignant tissue, which improves the drug–light interval and offers the clinicians a wider window of time in which they can irradiate the patient. Furthermore, a tumor-targeted delivery system diminishes the accumulation of PS in healthy tissues and, hence, decreases any adverse effects of photosensitivity. Tumor-targeting delivery systems of PS can help improve PDT by targeting specific tumor types, reaching inner organs more efficiently.

Much remains to be learned about PDT in cancer treatment. Therefore, further research is required for PDT to become the mainstream therapy preferred by oncologists and to ensure a hopeful future for the treatment of cancer.

## Figures and Tables

**Table 1:** Advantages and disadvantages of Photodynamic Therapy.

	Advantages	Disadvantages
PDT for cancer	-Fewer adverse effects -Little invasiveness -Short treatment time -Usable in outpatient settings -Double selectivity -Can be applied at the same location several times -Little or no scar after healing -Lower costs than other treatments	-Photosensitivity after treatment -Treatment efficacy depends on accurate light delivery to the tumor -Tissue oxygenation is crucial to the photodynamic effect -Impossible to treat metastatic cancers with current technology

## Abbreviations

The following abbreviations are used in this manuscript:

PDT	Photodynamic Therapy
PS(s)	Photosensitizer(s)
NP(s)	Nanoparticle(s)
PNPs	Polymeric Nanoparticles
SLNs	Solid Lipid Nanoparticles
MNs	Metallic nanoparticles
CDs	cyclodextrins
NLCs	nanostructured lipid carriers
AuNPs	Gold nanoparticles
ROS	Reactive oxygen species
MB	Methylene blue
HYP	Hypericin
m-THPC	meso-tetrakis (m-hydroxyphenyl) chlorin
LCs	liquid crystals
SiNPs	silica nanoparticles
IONPs	iron oxide nanoparticles
MEs	microemulsions
